# Risk Scores for Cardiac Implantable Electronic Device Infection: Which One to Believe In?

**DOI:** 10.3390/jcm11216556

**Published:** 2022-11-04

**Authors:** Michele Malagù, Luca Donazzan, Andrea Capanni, Paolo Sirugo, Claudio Rapezzi, Matteo Bertini

**Affiliations:** 1 Cardiology Unit, Azienda Ospedaliero-Universitaria di Ferrara, Via Aldo Moro 8, 44124 Ferrara, Italy; 2 Cardiology Unit, Ospedale di Bolzano, 39100 Bolzano, Italy

**Keywords:** cardiac device, CIED, infection, endocarditis, pacemaker, ICD, CRT, risk, score, predictor, arrhythmias, prognosis

## Abstract

Infections are important complications of cardiac implantable electronic devices (CIED), with a high prognostic impact. Several risk factors for CIED infections are known. Different studies have been published proposing different risk scores, in order to preoperatively assess the individual likelihood of developing a CIED infection. Among the different scores, large heterogeneity exists and there is no consensus or convergence on a single score finding large applicability in global practice. The aim of this review is to comprehensively present and analyze all the available risk scores for CIED infection, with particular regard to the evidence of comparison studies.

## 1. Introduction

Infection is the most feared complication in patients with cardiac implantable electronic devices (CIED), with an incidence of 1–3% during the lifetime and a mortality rate of up to 27.5% at three years [1,2,3,4].

Different strategies have been proposed to prevent infections. Strategies of proven efficacy include appropriate procedure timing, management of antithrombotic therapy, patient preparation, surgical technique, and adequate wound care [5,6]. However, the most important defense against CIED infection (and the most studied in more than 40 years of clinical trials) is systemic antibiotic prophylaxis [5,7]. In short, preoperative administration of antibiotics is clearly beneficial and represents the standard of care for all patients, recommended by international consensus, mostly with drugs covering *Staphylococcus aureus* species, such as beta-lactams or glycopeptides [5].

Further strategies have been proposed and showed benefits in the reduction of CIED infections, especially for patients at high risk. The absorbable antibacterial envelope significantly reduced CIED infections in the randomized WRAP-IT trial [8]. However, the wide adoption of absorbable envelopes is limited by costs, and, especially in Europe, its cost-effectiveness is favorable only in patients with a specific risk profile [9]. Prolonged antibiotic prophylaxis has been proposed but the reduction in CIED infection failed to reach statistical significance in an unselected “all comer” cohort of patients [10]. However, in selected patients at high risk, a more powerful antibiotic regimen may be beneficial [11].

Considering that 97–99% of patients are unlikely to develop a CIED infection during their lifetime, the proper identification of patients at high risk is crucial. Such a stratification of patients could be useful to guide individualized additional prophylactic strategies to those patients who may benefit from it. The risk factors for CIED infection are well known and have been evaluated in a multitude of studies [12]. Some of these factors are not modifiable, while others are [5]. Such a thorough understanding of risk factors led to the development of risk models in order to stratify patients, and different risk scores have been proposed. A reliable risk score could help clinicians to properly treat patients at risk and could be beneficial in terms of cost-effectiveness. However, large heterogeneity exists and there is no consensus or convergence on a single score finding large applicability in global practice.

The aim of this review is to comprehensively present the available risk scores for CIED infection and provide a critical evaluation of the pros and cons of each, with particular regard to the comparisons between them.

## 2. Risk Factors for CIED Infections

Many risk factors for CIED infection have been identified in a multitude of studies during the last 50 years [12]. These factors may be classified according to patient, procedure, and device characteristics. Factors related to the patient include age, male sex, diabetes mellitus, heart failure, renal insufficiency, chronic obstructive pulmonary disease, active neoplasia, fever within 24 h, anticoagulation, corticosteroids, central venous catheter, previous device infection, and trauma at the site of implant [3,11,12,13,14,15,16,17,18,19]. Factors related to the procedure are a lack of antibiotic prophylaxis, replacement, revision, upgrade, early reintervention, temporary pacing, procedure duration, operator experience, lead dislodgement, and hematoma [7,11,12,19,20,21,22,23]. Factors related to the device include implantable cardioverter–defibrillator (ICD) or cardiac resynchronization therapy (CRT), more than two leads, epicardial leads, and abdominal pockets [12,19,24]. These factors have been considered differently in the different scores that have been proposed.

## 3. Risk Scores

### 3.1. PADIT

The PADIT risk score was developed by Birnie et al. using the population of the PADIT trial and is currently the only risk score not derived from retrospective analysis [10,25]. For the score’s development, 200 samples were initially bootstrapped for internal validation. Independent predictors were identified using multivariable logistic prediction modeling. The performance of the full prediction model and risk score model was assessed in terms of calibration-in-the-large, calibration slope, and the C-statistic.

Five easy-to-access, independent predictors were recognized, namely prior procedures (P), age (A), depressed renal function (D), immunocompromised (I), and procedure type (T), giving a score ranging from 0 to 15 points. This classified patients into low (0 to 4), intermediate (5 to 6), and high (≥7) risk, with rates of hospitalization for infection of 0.51%, 1.42%, and 3.41%, respectively (Table 1).

It should be remarked that the subgroup analysis by PADIT infection risk score of the two antibiotic regimes (single dose or incremental) used in the PADIT trial showed no treatment effect (*p* for interaction = 0.37).

The risk score revealed high predictive power for reinfection, all-cause mortality, and hospitalization during the first year of follow-up, as well as cardiovascular mortality in patients submitted to lead extraction for CIED infection [26].

Moreover, the PADIT risk score showed a significant association with CIED infections, with overall modest prediction performance when tested in the RI-AIAC registry population [27]. There was no association with the occurrence of the composite clinical event of infection or all-cause death.

An independent validation of the score was performed in a data set extracted from U.S. healthcare claims by Ahmed et al [28]. In this population, the PADIT risk score served as a predictor of higher CIED infection risk. The risk of a major CIED infection increased by 28% for each one unit increase in PADIT risk score in a linear fashion. Furthermore, the authors suggest the use of prior CIED infection history to confer additional predictive value to the risk score.

### 3.2. SHARIFF

“SHARIFF” is a preoperative risk score developed to identify patients at high risk of CIED infection. It is calculated considering each of the following: diabetes mellitus, heart failure, oral anticoagulation, chronic corticosteroid use, renal insufficiency (serum creatinine >1.5 mg/dL), prior CIED infection, presence of more than two leads, presence of epicardial lead(s), temporary pacemaker at implantation, and replacement/upgrade procedure. Each factor counts for one point; therefore, the score ranges from 0 to 10. In the original study, a cohort of 1467 patients was retrospectively analyzed [19]. Occurrence of infection was compared between patients receiving an antibacterial envelope and a control group. At 6-month follow-up, a lower rate of infection was found in patients with SHARIFF score <3 (infection rate 1.0%) compared to those with SHARIFF score ≥3 (2.4%). A modified version of this score for first CIED implantation, evidently not considering prior CIED infection and replacement/upgrade, has been validated in a retrospective analysis of 1391 patients, in which a score ≥4 was an independent predictor of infection (relative risk 3.20, *p* = 0.029) [29]. The SHARIFF score was also used to stratify patient risk in the PRACTICE study, in which prolonged antibiotic prophylaxis was proposed for high-risk patients [11].

### 3.3. KOLEK

In 2013, Kolek et al. published a retrospective cohort study analyzing the outcomes of patients receiving an antibacterial envelope. The authors implanted the antibacterial envelope in patients considered at high risk for CIED infections, arbitrarily chosen as presenting at least two of the following: diabetes, renal insufficiency, anticoagulation, chronic corticosteroid use, fever or leukocytosis at the time of implantation, prior CIED infection, ≥3 leads, pacemaker dependency, or early pocket reentry. Patients with an antibacterial envelope were compared to a control group of patients, matched for the number of risk factors, with a CIED implanted before an antibacterial envelope became available. At a median follow-up of 18.7 ± 7.7 months, CIED infections were 20/899, 2.22%, significantly lower among patients receiving the antibacterial envelope compared to the control group (0.4% vs. 3%) [30].

In a subsequent study published by the same group, patients satisfying the presence of at least two of the same risk factors for CIED infections were divided into those receiving an absorbable antibacterial envelope (n = 135), those receiving a non-absorbable antibacterial envelope (n = 353), and those not receiving an antibacterial envelope (n = 636). The mean number of risk factors was 3.08 for the absorbable antibacterial envelope group, 3.20 for the non-absorbable antibacterial envelope group, and 3.09 for controls. The overall rate of CIED infection was 21/1124 (1.87%). Again, this study showed a lower rate of infection in patients treated with antibacterial envelopes also after a propensity score-matched cohort of either envelope or controls (0% vs. 2.8%) [31].

In both of these studies, patients were considered at high risk of infection if presenting at least two factors from the prespecified list. According to the study design, patients considered at low infective risk were not included.

### 3.4. MITTAL

In 2014, a study was published retrospectively evaluating 2880 consecutive patients undergoing a CIED procedure, divided into the pre-antibacterial envelope era and envelope era [32]. Infections necessitating the removal of the device were considered at a follow-up of 6 months. The “MITTAL” score was developed in order to stratify the risk of CIED infection: the investigators created a model of seven independent risk factors, with a point score assigned based on their weighting in the logistic regression model. The seven risk factors were the need for early pocket re-exploration, male sex, diabetes, the need for an upgrade procedure, congestive heart failure, arterial hypertension, and glomerular filtration rate <60 mL/min (Table 1). In the “pre-envelope era”, the infection rate was 1.0% in patients with a score of 0–7, 3.4% in patients with a score of 8–14, 11.1% in patients with scores >15. In the “envelope era”, 22% of patients (deemed at high risk) received the antibacterial envelope and the rate of infection was reduced to 0.7% and 0.0%, respectively, in patients with scores of 8–14 and 15–25.

Interestingly, the stratification of individual infective risk was used to determine which patients to treat with the antibacterial envelope, and this approach resulted in a significantly reduced rate of infection.

### 3.5. PACE DRAP

The PACE DRAP score was originally developed to estimate the risk of bleeding complications of CIED surgery among a cohort of 1100 consecutive patients [33]. Eight risk factors were identified at the multivariable analysis, corresponding to the acronym “PACE DRAP”: (P) presence of valvular prosthesis, (A) uncontrolled arterial hypertension (≥160/100 mmHg); (C) cancer (any malignancy diagnosed within the last 5 years); (E) elderly (≥75 years); (D) device type (CRT/ICD); (R) renal failure (glomerular filtration rate <60 mL/min/m^2^); (A) antiplatelets (clopidogrel, ticagrelor); and (P) procedure type (system upgrade, Table 2). In a subsequent analysis, a PACE DRAP score ≥6 was able to identify patients at high risk of CIED infection (sensitivity 72%, specificity 71%, positive predictive value 4.4%, negative predictive value 99.3%, area under curve 0.72) [34]. In the multivariable regression model, age >75 years, system upgrade procedure, duration of surgery >1 h, the presence of significant pocket hematoma, and early reintervention within 1 month of the primary procedure were identified as independent predictors of CIED infection (final model area under curve 0.95).

### 3.6. RI-AIAC

In 2022, two different scores were developed with the purpose of providing an assessment of both the risk of CIED infection and risk of CIED infection + all-cause mortality [27]. Eighteen Italian centers enrolled a total of 2675 patients, which were followed up for 12 months. The following risk factors were associated with the occurrence of CIED infection and included in the “RI-AIAC Infection score”: any CIED replacement, revision/upgrade/reimplantation, diabetes mellitus, hospital-acquired infection (Table 1). An RI-AIAC infection score ≥1 identified patients at higher risk of CIED infection (sensitivity 36%, specificity 90%) and was significantly associated with CIED infections (odds ratio (OR) 2.23, 95% confidence interval (CI) 1.02–4.85).

In parallel, the “RI-AIAC Event score” estimated the cumulative risk of CIED infection and all-cause death and was composed of the following: age, temporary pacing, renal failure, oral corticosteroids, hospital-acquired infection, and diabetes mellitus. An RI-AIAC event score ≥2 identified patients at higher risk of the composite clinical event (sensitivity 59%, specificity 69%).

Both scores were developed based on the multivariate logistic analysis of the study cohort and subsequently validated in an independent cohort of 1017 patients.

Table 1 summarizes the currently available infective risk scores and reports calculation details for every risk factor in each risk score.

## 4. Comparisons

Different scores report the same risk factor. The most represented are renal impairment (reported in 5 out of 6 risk scores with different definitions), procedure type (5/6 with different definitions), diabetes (4/6), immunocompromise or corticosteroid use (3/6), anticoagulation/antiplatelets (3/6), heart failure (2/6), age (2/6), early pocket reintervention (2/6), prior CIED infection (2/6), and pre-implant infection (2/6). Interestingly, the same risk factor accounts for different weights in the risk scores. For example, diabetes has one point weight in the SHARIFF, KOLEK and RI-AIAC scores, while it weighs three points in the MITTAL score. This makes the diabetic patient a high-risk patient in the RI-AIAC score, which is not true for the others. Moreover, older age gives a higher infective risk in the PACE-DRAP, while it seems to reduce the risk in the PADIT score. There is no clear reason for this, with older people having multiple comorbidities and risk factors for infection, but with different studies giving opposite evidence [12,35,36]. Furthermore, it has been hypothesized that a combination of factors, rather than merely the absolute number, could play a role [32].

It is possible that the type of procedure performed in PACE-DRAP (ICDs only) and PADIT (both PMs and ICDs) could also have an impact. Very elderly and frail patients are not eligible for ICD implantation and were excluded from both populations. In PADIT, the risk of infection was significantly lower for new pacemaker or pacemaker replacement procedures, which are more often performed in elderly people. This was also true in a Danish prospective pacemaker registry of more than 46,000 patients [3].

The PADIT and SHARIFF risk scores were unable to distinguish patients who would benefit from more intensive antibacterial therapy. On the contrary, scores applied to an “antibacterial envelope strategy” (i.e., local antibacterial delivery via antibacterial envelope in addition to systemic antibacterial therapy), such as the KOLEK and MITTAL scores, identified patients who would benefit the most from this additional antibiotic therapy. The PACE-DRAP and RI-AIAC scores were not tested in association with different antibacterial therapies to explore their efficacy in therapy guidance.

The original studies proposing the different scores presented noteworthy differences (Table 2). First of all, the PADIT trial enrolled 19,603 patients, a number much higher than all the other studies, which ranged from 899 (KOLEK) to 2675 (RI-AIAC) and 2880 (MITALL) [25,27,30,32]. The PADIT, SHARIFF, and RI-AIAC scores have been validated in external cohorts. However, for the PADIT trial, the external validation cohort was particularly large, evaluating a data set of 51,623 patients from the healthcare claims [28].

Despite the development of several scores, only a few comparisons between them have been published. In 2020, a study by Slawek-Szmyt et al. aimed to evaluate the utility of the PADIT and PACE DRAP scores to predict CIED infection [34]. One thousand patients undergoing ICD or CRT implantation/replacement/upgrade were prospectively enrolled and followed-up for 12 months, with an incidence of CIED infection of 1.8%. Logistic regression analyses were used to identify the independent predictors of infection, and receiver operating characteristic curve (ROC) analysis was performed to determine the predictive value of the two scores and for the evaluation of the regression models. The following characteristics were identified as independent predictors of CIED infection in the multivariable regression model: age >75 years (OR 5.93, CI 1.77–19.84), system upgrade procedure (OR 6.46, CI 1.94–21.44), duration of surgery >1 h (OR 13.96, CI 4.40–44.25), pocket hematoma (OR 4.95, CI 1.62–15.13), and reintervention within 1 month (OR 16.29, CI 3.14–84.50). The PACE DRAP score better discriminated between patients with high and low risk of infection (AUC 0.72), in comparison with the PADIT score (AUC 0.63). Furthermore, the two scores had similar specificity (PADIT 76.3%, PACE-DRAP 71.1%), but the PACE-DRAP showed higher sensitivity (50% vs. 72.2%).

In 2022, Boriani et al., in the abovementioned study that presented and validated the RI-AIAC score, also provided a comparison between the newly proposed one and the pre-existing PADIT, KOLEK, and SHARIFF scores [27]. Interestingly, in the study cohort, only the PADIT and RI-AIAC infection scores were significantly associated with CIED infection (C-index 0.64 for both, *p* = 0.010 and 0.015, respectively), while KOLEK and SHARIFF were not (C-index 0.56 and 0.58, *p* = 0.261 and 0.159, respectively). After adjusted regression analysis, the RI-AIAC infection score showed the strongest association with the outcome (OR 2.38, 95% CI 1.6–3.55 for each point), with the PADIT revealing less power (OR 1.28, 95% CI 1.1–1.5). However, in the external validation cohort of 1017 patients, none of the four scores was able to predict infections (PADIT C-index 0.53, *p* = 0.746, KOLEK C-index 0.64, *p* = 0.065, SHARIFF C-index 0.62, *p* = 0.131, RI-AIAC infection C-index 0.58, *p* = 0.292, Table 2).

## 5. Conclusions

Several risk scores have been proposed to predict CIED infections. Only some of the risk factors are common to different scores but have different definitions and weight. All the available scores are of easy application and can be calculated quickly based on medical history, common laboratory tests, and procedure type. Among the different scores, the PADIT has been validated in many more patients but, when compared to others, it has been proven less powerful than PACE DRAP, KOLEK, SHARIFF, and RI-AIAC. However, the available comparisons did not comprehensively consider all the available scores. Above all, according to the results, the predictive power of each score is low. Further studies are needed.

## Figures and Tables

**Table 1:** Summary of risk scores for cardiac implantable electronic device infections.

INFECTIVE RISK SCORE	RISK FACTORS					POINTS	SCORE	INFECTION RISK
PADIT	Age			<60 years		2	0–4	Low (<1%)
				60–69 years		1		
	Renal insufficiency (eGFR <30 mL/min)					1		
	Immunocompromised *					3	5–6	Intermediate (1–3%)
	Procedure type		ICD			2		
			CRT			4		
			Revision/Upgrade			5	≥7	High (>3%)
	Number of previous procedures				1	1		
					≥2	4		
SHARIFF	Diabetes					1	<3	Low (1%)
	Heart failure					1		
	Oral anticoagulation					1		
	Chronic corticosteroid use					1		
	Renal insufficiency (Cr > 1.5 mg/dL)					1		
	Prior CIED infection					1	≥3	High (2.4%)
	>2 leads					1		
	Epicardial lead(s)					1		
	Temporary pacing					1		
	Generator replacement or upgrade					1		
KOLEK	Diabetes					1	<2	Low
	Renal insufficiency (Cr ≥1.5 mg/dL)					1		
	Systemic anticoagulation					1		
	Chronic corticosteroid use					1		
	Preimplant fever ⁺ or leukocytosis †					1		
	Prior CIED infection					1	≥2	High (1.9–2.2%)
	≥3 transvenous leads					1		
	Pacemaker dependence					1		
	Early pocket reentry (within 2 weeks of implantation)					1		
MITTAL	Early pocket reintervention					11	0–7	Low (1%)
	Male sex					6		
	Diabetes					3	8–14	Intermediate (3.4%)
	Upgrade					2		
	Heart failure					1		
	Hypertension					1	≥15	High (11.1%)
	Renal dysfunction (eGFR < 60 mL/min)					1		
PACE DRAP	Valvular prosthesis					2	<6	Low (0.7%)
	Hypertension (≥160/100 mmHg)					2		
	Cancer (within last 5 years)					2		
	Age ≥ 75 years					2		
	CRT/ICD surgery					2		
	Upgrade					2	≥6	High (4.6%)
	Antiplatelets	Clopidogrel				2		
		Ticagrelor				3		
	Renal disfunction (eGFR < 60 mL/min)					1		
RI AIAC	Revision/Upgrading/Reimplantation					2	0	Low
	CIED replacement					1		
	Diabetes					1	≥1	High
	Hospital-acquired infection					1		

* Immunocompromised is defined as receiving therapy that suppresses resistance to infection (e.g., immunosuppression, chemotherapy, radiation, long-term or recent high-dose steroids) or having a disease that is sufficiently advanced to suppress resistance to infection (e.g., leukemia, lymphoma, HIV infection). ⁺ ≥100.5 F. † ≥11,000 white blood cells/μL within 24 h prior to implantation. eGFR: estimated glomerular filtration rate; ICD: implantable cardioverter–defibrillator; CRT: cardiac resynchronization therapy; Cr: creatinine; CIED: cardiac implantable electronic device.

**Table 2:** Differences in original studies proposing risk scores for CIED infection and validation cohorts and comparisons.

	PADIT	SHARIFF	KOLEK	MITTAL	PACE DRAP	RI AIAC
Reference	[25]	[19]	[30]	[32]	[34]	[27]
Design	Prospective, multicenter, cluster-randomized	Retrospective, single-center	Prospective, single-center	Retrospective, single-center	Prospective, single-center	Prospective, multicenter
Patients	19,603	1476	899	2891	1000	2675
Follow-up	1 year	6 months	18.7 ± 7.7 and 42.4 ± 5.2 months	6 months	1 year	1 year
Infection rate	0.9%	1.29%	2.22%	1.14%	1.8%	1.1%
External validation	Yes [28]	Yes [29]	Yes [31]	No	No	Yes [27]
Patients in external validation cohort	51623	1391	1124	//	//	1017
AUC in PACE DRAP study comparison [34]	0.63	//	//	//	0.72	//
C-index (95% CI) in RI-AIAC study comparison [27]	0.53 (0.38–0.67)	0.62 (0.46–0.77)	0.64 (0.5–0.79)	//	//	0.58 (0.42–0.74)

AUC: area under the curve; CI: confidence interval; //: no external validation.
